# Making health economic models Shiny: A tutorial

**DOI:** 10.12688/wellcomeopenres.15807.2

**Published:** 2020-07-31

**Authors:** Robert Smith, Paul Schneider

**Affiliations:** 1 School of Health and Related Research, University of Sheffield, Regents Court, Sheffield, S1 4DA, UK

**Keywords:** Health Economics, R, RShiny, Decision Science

## Abstract

Health economic evaluation models have traditionally been built in Microsoft Excel, but more sophisticated tools are increasingly being used as model complexity and computational requirements increase. Of all the programming languages, R is most popular amongst health economists because it has a plethora of user created packages and is highly flexible. However, even with an integrated development environment such as R Studio, R lacks a simple point and click user interface and therefore requires some programming ability. This might make the switch from Microsoft Excel to R seem daunting, and it might make it difficult to directly communicate results with decisions makers and other stakeholders.

The R package Shiny has the potential to resolve this limitation. It allows programmers to embed health economic models developed in R into interactive web browser based user interfaces. Users can specify their own assumptions about model parameters and run different scenario analyses, which, in the case of regular a Markov model, can be computed within seconds. This paper provides a tutorial on how to wrap a health economic model built in R into a Shiny application. We use a four-state Markov model developed by the Decision Analysis in R for Technologies in Health (DARTH) group as a case-study to demonstrate main principles and basic functionality.

A more extensive tutorial, all code, and data are provided in a GitHub repository.

## Introduction

As the complexity of health economic decision models increase, there is growing recognition of the advantages of using high level programming languages (e.g. R, Python, C++, Julia) to support statistical analysis. Depending on the model that is being used, Microsoft Excel can be relatively slow. Certain types of models (e.g. individual-level simulations) can take a very long time to run or become computationally infeasible, and some essential statistical methods can hardly be implemented at all (e.g. survival modelling, network meta-analysis, value of sample information), or rely on exporting results from other programs (e.g. R, STATA, WinBUGs).

Of all the high level programming languages, R is the most popular amongst health economists ^1^. R is open source and supported by a large community of statisticians, data scientists and health economists. There are extensive collections of (mostly free) online resources, including packages, tutorials, courses, and guidelines. Chunks of code, model functions, and entire models are shared by numerous authors, which allow R users to quickly adopt and adapt methods and code created by others. Importantly for the UK, R is also currently the only programming environment accepted by NICE for HTA submissions, the alternative submission formats Excel, DATA, Treeage, and WinBUGs are all software applications ^2^.

Despite the many strengths of the script based approach (e.g R) to decision modelling, an important limitation has been the lack of an easy-to-understand user-interface, which would be useful as it "facilitates the development and communication of the model structure" (p.743) ^1^. While it is common practice for ’spreadsheet models’ to have a structured front tab, which allows decision makers to manipulate model assumptions and change parameters to assess their impact on the results, up until recently, R models had to be adapted within script files or command lines.

Released in 2012, Shiny is an R-package that can be used to create a graphical, web browser based interface. The result looks like a website, and allows users to interact with underlying R models without the need to manipulate the source code ^3^. Shiny has already been widely adopted in many different areas and by various organisations to present the results of statistical analysis ^4^. Within health economics Shiny is currently being used to conduct network meta analysis ^5^ and value of information analysis ^6, 7^.

Using Shiny, it is possible to create flexible user interfaces that allow users to specify different assumptions, change parameters, run underlying R code and visualise results. The primary benefit of this is that it makes script based computer models accessible to those with no programming knowledge - opening models up to critical inquiry from decision makers and other stakeholders ^8^. Other benefits come from leveraging the power of R’s many publicly available packages; for example, allowing for publication quality graphs and tables to be downloaded, user specific data-files to be uploaded, open-access data to be automatically updated and, perhaps most importantly, to efficiently run comprehensive probabilistic sensitivity analyses in a fraction of the time that it would take in Microsoft Excel. Shiny web applications for R health economic decision models seem particularly useful in cases where model parameters are highly uncertain or unknown, and where analysis is conducted with heterogeneous assumptions (e.g. for different populations). Examples of well-designed shiny applications include, for example, the the Innovation and Value Initiative's open-source rheumatoid arthritis individual patient simulation model, Bresmed’s ‘IntRface’ application, and the SHARP CKD-CVD outcomes model ^9– 11^.

While, from a transparency perspective, it is preferable that models constructed in R are made open-access to improve replicability and collaboration, it is not a requirement ^12^. Sensitive and proprietary data and/or models can be shared internally, or through password-protected web applications, negating the need to email zipped folders. Once an R model and a Shiny application have been created, they can also be easily adapted, making it possible to quickly update the model when new information becomes available. Several authors have postulated that there is considerable potential in using Shiny to support and improve health economic decision making. Incerti *et al.* (2019) identified web applications as being an essential part of modelling, stating that they "believe that the future of cost-effectiveness modeling lies in web apps, in which graphical interfaces are used to run script-based models" (p.577) ^13^. Similarly, Baio and Heath (2017) predicted that R Shiny web apps will be the "future of applied statistical modelling, particularly for cost-effectiveness analysis" (p.e5) ^14^. Despite these optimistic prognoses, adoption of R in health economics has been slow and the use of Shiny seems to have been limited to only a few cases. A reason for this might be the lack of accessible tutorials tailored towards an economic modeller audience.

Here, we provide a simple example of a Shiny web app, using a general four-state Markov model. The model is based on the ’Sick-Sicker model’, which has been described in detail in previous publications ^15, 16^ and in open source teaching materials by the DARTH workgroup ^17^. The model was slightly adapted to implement probabilistic sensitivity analysis. This paper aims to provide a tutorial, designed specifically for those familiar with decision modelling in R, to create web-based user interfaces for R models using R Shiny.

## Methods

While the focus of this tutorial is on the application of Shiny for health economic models, below we provide a brief overview of the "Sick-Sicker model". For further details, readers are encouraged to consult previous publications by the DARTH group ^15, 16, 18^ and the DARTH group website ^17^.

The Sick-Sicker model is a four-state (Healthy, Sick, Sicker or Dead) time-independent Markov model. The cohort progresses through the model in cycles of equal duration, with the proportion of those in each health state in the next cycle being dependant on the proportion in each health state in the current cycle and a time constant transition probability matrix.

The analysis incorporates probabilistic sensitivity analysis (PSA) by creating a data-frame of PSA inputs (one row being one set of model inputs) based on cost, utility and state transition probability distributions using the function *f_gen_psa* and then running the model for each set of PSA inputs using the model function *f_MM_sicksicker*. We therefore begin by describing the two functions *f_gen_psa* and *f_MM_sicksicker* in more detail before moving on to demonstrate how to create a user-interface. In this tutorial, we follow Alarid-Escudero *et al.’s* (2019) coding framework and add to it the prefix 'f_' to denominate functions ^15^.

## Functions

The *f_gen_psa* function (see the file f_gen_psa.R in the open access repository: https://doi.org/10.5281/ zenodo.3727052 ^19^) returns a data-frame of probabilistic sensitivity analysis inputs: transition probabilities between health states using a beta distribution, hazard rates using a log-normal distribution, costs using a gamma distribution and utilities using a truncnormal distribution. It relies on two inputs, the number of simulations (PSA inputs), and the cost (which takes a fixed value). We set the defaults to 1000 and 50, respectively.

**Running the model for a specific set of PSA inputs**

The function *f_MM_sicksicker* (see the file f_MM_sicksicker in the open access repository: https://doi.org/10.5281/zenodo.3727052) makes use of the *with* function, which applies an expression (in this case the rest of the code) to a data-set (in this case params, which will be a row of PSA inputs).

The function first calculates transition probabilities from each health state to each health state and uses these to fill a transition probability matrix ( *m_P*). It then creates a matrix for the markov trace ( *m_TR*) which has t+1 nrows and four columns (one for each health state). The ‘PROCESS’ part of the code then ‘loops’ through the markov model, using matrix multiplication (elicited using %*% in R), iteratively computing, for each period, the proportion of the population that is in each state.

In the ‘OUTPUT’ section of the code the markov trace ( *m_TR*) is multiplied (again using matrix multiplication) with vectors of health state utilities (e.g. v_u_trt) and costs (e.g. v_c_trt), giving a vector of total costs and utilities in each time interval. These vectors are then discounted using a discount weight vector (e.g. *v_dwe* & *v_dwe*) to arrive at a single cost/QALY value. The resulting total discounted costs and QALY estimates for the treatment and the no-treatment group then are combined into a vector and returned from the function. In this simple example, treatment only influences utilities and costs, not transition probabilities. For further details on the underlying model, we refer to the published source code ^19^

**Creating the model wrapper**

When using a web application, it is likely that the user will want to be able to change parameter inputs and rerun the model. In order to make this simple, we recommend wrapping the entire model into a function. We call this function *f_wrapper*, using the prefix *f_* to denote that this is a function.

The wrapper function has as its inputs all the parameters that we may wish to vary using R-Shiny. We set the default values to those of the base model in any report/publication. The model then generates PSA inputs using the *f_gen_psa* function, creates an empty table of results, and runs the model for each set of PSA inputs (a row from *df_psa*) in turn. The function then returns the results in the form of a data-frame with n=5 columns and n=psa rows. The columns contain the costs and QALYs for treatment and no treatment for each PSA run, as well as an ICER for that PSA run.

**Model wrapper function**



f_wrapper<—function(

#——  User adjustable inputs ——#

#age at  baseline
n_age_init = 25,
#maximum age of follow up
n_age_max = 110,
#discount rate for costs and QALYS
d_r     = 0.035,
#number of simulations
n_sim   = 1000,
#cost of intervention treatment in states sick and sicker
c_Trt   = 50

){


#—— Unadjustable inputs ——#

#number of cycles
n_t<— n_age_max— n_age_init
#the 4 health states of the model:
v_n <—c("H","S1","S2","D")
#number of health states
n_states<—length(v_n)

#—— Create PSA Inputs ——#

df_psa <— f_gen
                    _psa(n_sim= n_sim,
c_Trt =c_Trt)

#——  Run  PSA ——#

#Initialize  matrix of results outcomes
m_out <—matrix(NaN,
nrow= n_sim,
ncol= 5,
dimnames=list(1:n_sim,
c("Cost_NoTrt",  
                    "Cost_Trt",
"QALY_NoTrt","QALY_Trt",
"ICER")))
#run model for each row of PSA inputs
for(i in 1:n_sim){

#store results in row of results matrix
m_out[i,] <— f_MM_sicksicker(df_psa[i, ])

}#close model loop


#—— Return results ——#

#convert matrix to dataframe (for plots)
df_out <— as.data.frame(m_out)

#output the dataframe from the function
return(
                    df_out)
}#end of function



## Integrating into R-Shiny

The next step is to integrate the model function into a Shiny web-app. This is done within a single R file, which we call *app.R*. This can be found within the GitHub repository here.

The app.R script has three main parts, each are addressed in turn below:

- set-up (getting everything ready so the user-interface and server can be created)
- user interface (what people will see)
- server (R code running in the background)

**Figure 1.**
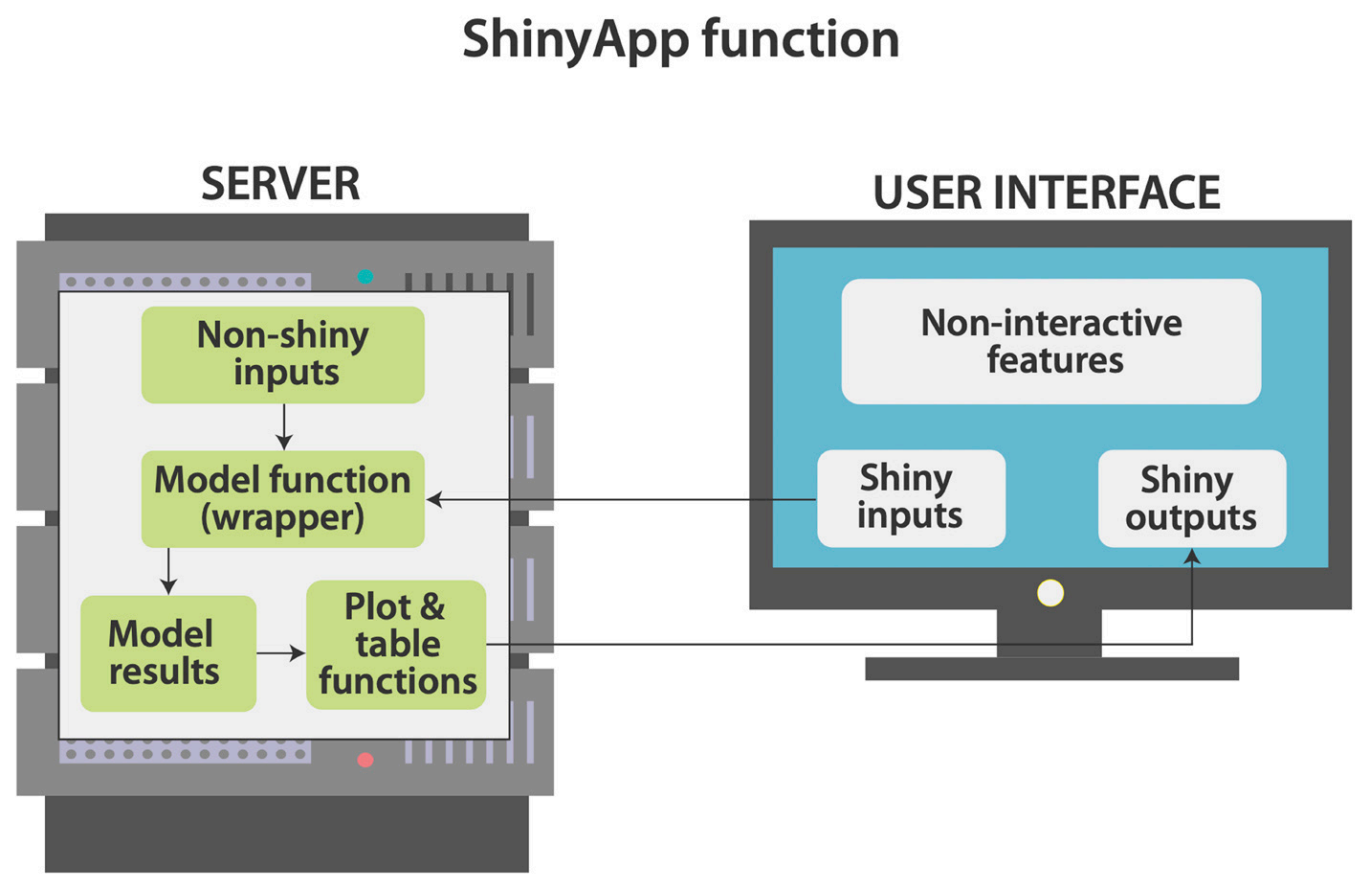
Diagram depicting how the Sick-Sicker app is structured.

Figure 1 depicts the relationship between the server and the user interface within the Shiny application. On a conceptual level, the user interface has three components: Shiny inputs (objects that the user can specify, e.g. by inputting a number), Shiny outputs (objects created on the server side, e.g. plots and tables), and non-interactive features (any fixed elements, such as texts, headings, logos etc.). The server works almost like a normal R session. It runs various R operations, including the model function, which takes non-Shiny inputs (defined only on the server side) and some Shiny inputs from the user interface. The results are then sent to the user interface and displayed as Shiny outputs.

**Initial set-up**

The set-up is relatively simple. First, load the R-Shiny package from your library so that you can use the *shinyApp* function. The next step is to use the *source* function in baseR to run the script that creates the *f_wrapper* function, being careful to ensure your relative path is correct (’./wrapper.R’ should work if the wrapper.R file is in the same folder as the app.R file).

**Code initialization (within app.R)**



# install 'shiny' if haven't already.
## install.packages("shiny")  # necessary if you don't already have the function 'shiny' installed.

## we need the function shiny installed, this loads it from the library.

# source the wrapper function.
source(
                    "./wrapper.R")


**Creating the user interface function**

The user interface is extremely flexible, we show the code for a very simple structure (fluidpage) with a sidebar containing inputs and a main panel containing outputs. We have done very little formatting in order to minimize the quantity of code while maintaining basic functionality. In order to get an aesthetically pleasing application, we recommend much more sophisticated formatting, relying on CSS, HTML and Javascript.

**Figure 2.**
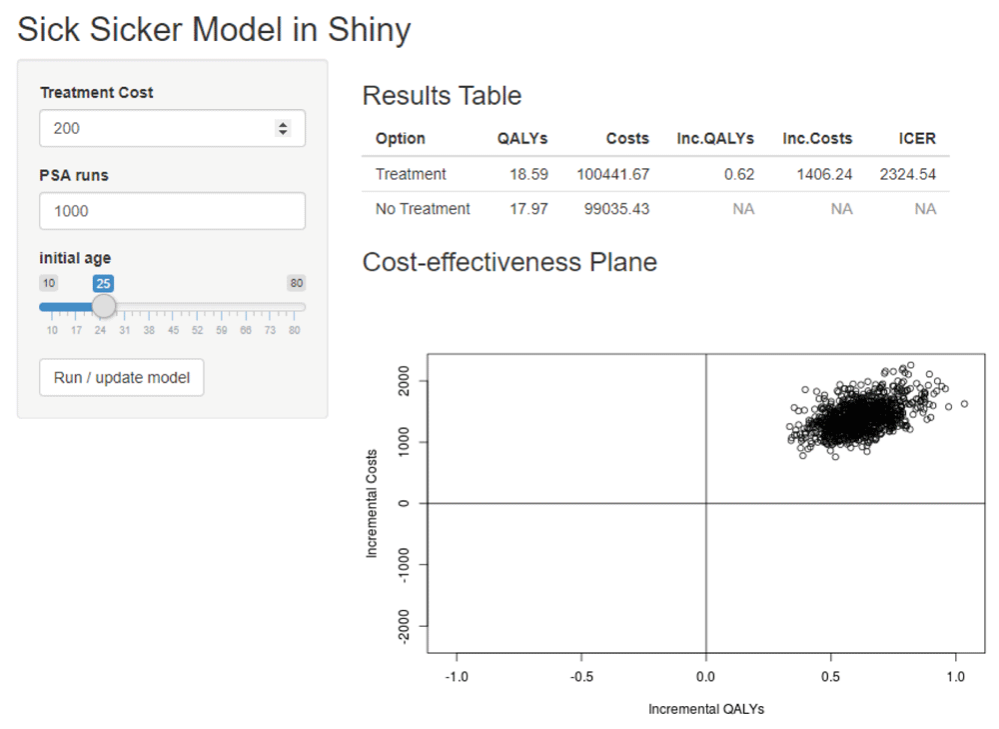
Screen-print of Sick-Sicker model user interface.

The example user interface displayed in Figure 2 and online on this website. The user interface is a *fluidpage* in a *sidebarLayout* (other types of layout are available). The *sidebarLayout* is made up of two components, a titlepanel and a sidebar layout display (which itself is split into a sidebar and a main panel). This is a basic structure used for teaching purposes, there are a plethora of templates available online.

The title panel contains the title “Sick Sicker Model in Shiny”, the sidebar panel contains two numeric inputs and a slider input (“Treatment Cost”, “PSA runs”, “Initial Age”) and an action button (“Run / update model”). The values of the inputs have ID tags (names), which are recognised and used by the server function, we denote these with the prefix “SI” to indicate they are ’Shiny Input’ objects ( *SI_c_Trt*, *SI_n_sim*, *SI_n_age_init*). The action button also has an ID, this is not an input into the model wrapper *f_wrapper* so we leave out the SI and call it *run_model*.

The main panel contains two objects that have been output from the server: *tableOutput(“SO_icer_table”)* is a table of results, and *plotOutput(“SO_CE_plane”)* is a cost-effectiveness plane plot. It is important that the format (e.g. *tableOutput*) matches the format of the object from the server (e.g. *SO_icer_table*). Again, the *SO* prefix reflects the fact that these are Shiny Outputs. The two *h3()* functions are simply headings, which appear as “Results Table” and “Cost-effectiveness Plane”.

**Shiny user interface code**


ui <— fluidPage (#creates empty page

# title of app
titlePanel("Sick Sicker Model in Shiny"),
#layout is a sidebar—layout
sidebarLayout(
sidebarPanel(#open sidebar panel
#input type numeric
numericInput(inputId ="SI_c_Trt",label ="Treatment Cost",
value = 200,
min= 0,
max= 400),
numericInput(inputId ="SI_n_sim",
label ="PSA runs",
value = 1000,
min= 0,
max= 400),
#input type slider
sliderInput(inputId ="SI_n_age_init",
label ="Initial Age",
value = 25,
min= 10,
max= 80),
#action button runs model when pressed
actionButton(inputId ="run_model",
label   ="Run model")
),#close sidebarPanel
#open main panel
mainPanel(
#heading (results table)
h3("Results Table"),
#tableOutput id = icer_table, from server
tableOutput(outputId ="SO_icer_table"),
#heading (Cost effectiveness plane)
h3("Cost—effectiveness Plane"),
#plotOutput id = SO_CE_plane, from server
plotOutput(outputId ="SO_CE_plane")
)#close mainpanel
)#close side barlayout
)#close UI fluidpage


**Creating the server function**

The server is marginally more complicated than the user interface. It is created by a function with inputs and outputs. The observe event indicates that when the action button *run_model* is pressed the code within the curly brackets is run. The code will be re-run if the button is pressed again. Setting the parameter ignoreNULL to False lets the model run when it is initialised, i.e. when the app is started.

The first thing that happens when the *run_model* button is pressed is that the model wrapper function *f_wrapper* is run with the user interface inputs ( *SI_c_Trt*, *SI_n_age_init*, *SI_n_sim*) as inputs to the function. The *input* prefix indicates that the objects have come from the user interface. The results of the model are stored as the data-frame object *df_model_res*.

The ICER table is then created and output (note the prefix *output*) in the object *SO_icer_table*. The function *renderTable* generates a table from the model results to display it on the web interface. See previous section on the user interface and note that the *tableOutput* function has as an input *SO_icer_table*. The function *renderTable* rerenders the table continuously so that the table always reflects the values from the data-frame of results created above. In this simple example we have created a table of results using code within the script. Normally we would use a custom function that creates a publication quality table that is aesthetically pleasing. There are different packages that provide this functionality ^15, 20, 21^.

The cost-effectiveness plane is created in a similar process, using the *renderPlot* function to continuously update a plot, which is created using baseR plot function using incremental costs and QALYs calculated from the results dataframe *df_model_res*. For aesthetic purposes we recommend this is replaced by a ggplot2 or plotly plot, which have much improved functionality ^22, 23^. As with the results table, there are also numerous health economic modelling specific R packages that have plotting features ^15, 20, 21^.

**Shiny server function**


server <—function(input, output){

#when action button pressed ...
observeEvent(input$run_model,
ignoreNULL = F, {

#Run  model function with Shiny inputs
df_model_res = f_wrapper(
c_Trt = input$SI_c_Trt,
n_age_init = input$SI_n_age_init,
n_sim = input$SI_n_sim)

#—— CREATE COST EFFECTIVENESS TABLE ——#

#renderTable continuously updates table
output$SO_icer_table <— renderTable({
df_res_table <—data.frame( #create dataframe

Option = c("Treatment","No Treatment"),

QALYs  = c(mean(df_model_res$QALY_Trt),
mean(df_model_res$QALY_NoTrt)),
Costs  = c(mean(df_model_res$Cost_Trt),
mean(df_model_res$Cost_NoTrt)),
Inc.QALYs = c(mean(df_model_res$QALY_Trt) —
mean(df_model_res$QALY_NoTrt),
               NA),
 
 Inc.Costs = c(mean(df_model_res$Cost_Trt) —
mean(df_model_res$Cost_NoTrt),
               NA),

 ICER = c(mean(df_model_res$ICER), NA)
 
 ) #close data—frame




#round the data—frame to two digits
df_res_table[,2:6] = round(
        
                    df_res_
                    table[,2:6],digits = 2)

#print the results table
df_res_table
   
  }) 
                    #table plot end.
#—— CREATE COST EFFECTIVENESS PLANE ——#

#render plot repeatedly updates.
output$SO_CE_plane <— renderPlot({

#calculate incremental costs and qalys
df_model_res$inc_C<—df_model_res$Cost_Trt —
df_model_res$Cost_NoTrt

df_model_res$inc_
                    Q<—df_model_res$QALY_Trt —
df_model_res$QALY_NoTrt

#create cost effectiveness plane plot

plot(

                    #x y are incremental QALYs Costs
x =df_model_res$inc_Q,

                    y =df_model_res
                    $inc_C,

#label axes
xlab = 
                    "Incremental QALYs",
ylab = 
                    "Incremental Costs",

#set x—limits and y—limits for plot.
xlim =c(min(df_model_res
                    $inc_Q,
df_model_res$inc_
                    Q*—1),
max(df_model_res
                    $inc_Q,
df_model_res$inc_Q*—1)),

ylim =c(min(df_model_res$inc_
                    C,
              
                    df_model_res
                    $inc_C*—1),
max(df_model_res
                    $inc_
                    C,
              
                    df_model_res$inc_C*—1)),

#include y and y axis lines .
abline(h = 0,v = 0)
)#CE plot end
})#renderplot end
})#Observe event end
}#Server end


**Running the app**

The app can be run within the R file using the function *shinyApp*, which depends on the *ui* and *server* that have been created and described above. Running this creates a Shiny application in the local environment (e.g. your desktop). It is also possible to deploy the application onto the web from RStudio using the shinyapps.io server (using the publish button in the top right corner of the R-file in R-Studio). Alternatively, apps can be hosted on private servers and integrated into existing websites. Server specifications should be chosen to match model requirements: while simple Markov chain state transition models may run on almost any server, more computationally burdensome models (e.g. agent-based models) may require considerable computing power. A step by step guide to the process of publishing applications can be found on the R-Shiny website or other online resources ^3, 24^.

**Running the app**


shinyApp(ui , server)



**Additional functionality**

The example Sick-Sicker web-app that has been created is a simple, but functional, R-Shiny user interface for a health economic model. There are a number of additional functionalities, many of which are covered in an online book by Hadley Wickham ^24^.

- fully customised user interface aesthetics. Since the user interface is translated into HTML and CSS it is possible to customise all components (such as colors, fonts, graphics, layouts and backgrounds) ^3, 25^.
- leverage many popular R packages to visualise model inputs (e.g. distributions) and outputs (e.g. plots and results tables) ^22, 23, 26^.
- upload files containing input parameters and data to the app ^24^.
- download specific figures and tables from the app ^24^.
- create a downloadable full report including model inputs and outputs ^24^.
- send model results/report to an email address once the model has finished running ^27^.

It is also possible to integrate all of the steps of health economic evaluation into one program. After selecting a subgroup of studies to use as inputs for a network meta-analysis, and economic model assumptions, the user would be required to simply click a ’run’ button. They would then be presented with results of the network meta-analysis, economic model and value of information analysis in one simple user-interface. The app user would then also be able to download a report (or have it sent to an email address) with the model results and appropriate visualisations updated to reflect their assumptions.

## Discussion

In this paper, we demonstrated how to generate a user-friendly interface for an economic model programmed in R, using the Shiny package. This tutorial shows that the process is relatively simple and requires limited additional programming knowledge than that required to build a decision model in R.

The movement towards script based health economic models with web based user interfaces is particularly useful in situations where a general model structure has been created with a variety of stakeholders in mind, each of which may have different assumptions (input parameters) and wish to conduct sensitivity analysis specific to their decision. For example, the World Health Organisation Department of Sexual and Reproductive Health and Research recently embedded a Shiny application into their website ^28^. The application runs a *heemod* model ^20^ in R in an external server, and allows users to select their country and specify country specific assumptions (input parameters), run the model and display results.

A well designed user interface can allow users to explore and better understand the relationship between model input and results. This allows users to tailor the health economic model to their specific situation and assumptions, without the expense of creating a new model. This may be particularly useful in the following scenarios: Firstly, in areas where one health economic decision model is applied in range of circumstances (e.g. in public health, models are often built to be used in a number of different countries). Secondly, when the full model source code can or may not be shared (e.g. for proprietary or privacy reasons). R-Shiny apps can be made available in a way that allows users to interact with the web interface, without revealing the model behind it. Finally, R shiny apps may enable stakeholders and decision makers, who would otherwise not be able to interact directly with statistical computer models, to experiment with and to reflect on various scenarios and the validity of model inputs and outputs.

However, in all of these scenarios, the ability for users to test different assumptions is not without limits: the available options to vary point estimates or the uncertainty around input parameters are defined by the model developer, and it is also not possible to specify alternative model structures or test any other aspect that the developer did not implement. Therefore, the model source code and data will still need to be made available to reviewers to allow for a thorough assessments of health economic models. Further investigation into how to communicate economic decisions models in a transparent and inclusive way is an important avenue of future research. 

The authors’ experience of creating user-interfaces for decision models has led to the conclusion that the most efficient method is to work iteratively, starting with a very simple working application, and adding functionality step by step, testing the app at each iteration to ensure it works as intended. It is worth noting that the simple model chosen as an exemplar is a markov model, however the method described can be applied to any model built using R, regardless of model type. For example, in this case, the *f_MM_sicksicker* function could also be replaced by a function containing any other type of model (e.g. a DES model).

There are several challenges that exist with the movement toward script based models with web-based user-interfaces. The first is the challenge of up-skilling health economic modellers used to working in Microsoft Excel. We hope that this tutorial provides a useful addition to previous tutorials demonstrating how to construct decision models in R ^16^. A second, and crucial challenge to overcome, is a concern about deploying highly sensitive data and methods to an external server. While server providers such as ShinyIO provide assurances of SSR encryption and user authentication clients with particularly sensitive data may still have concerns. This problem can be avoided in two ways: firstly, if clients have their own server and the ability to deploy applications they can maintain control of all data and code, and secondly, the application could simply not be deployed, and instead simply created during a meeting using code and data shared in a zip file. Finally, a challenge (and opportunity) exists to create user-interfaces that are most user-friendly for decision makers in this field; this is an area of important research that requires closer collaboration between decision makers, stakeholders and health economic decision model developers.

## Conclusion

The creation of web application user interfaces for health economic models constructed in high level programming languages should improve their usability, allowing stakeholders and third parties with no programming knowledge to conduct their own sensitivity analysis remotely. This tutorial provides a reference for those attempting to create a user interface for a health economic decision model created in R. Further work is necessary to better understand how to design interfaces that best meet the needs of different decision makers.

## Data availability

All data underlying the results are available as part of the article and no additional source data are required.

## Software availability

Source code available from: https://github.com/ RobertASmith/paper_makeHEshiny

Archived source code at time of publication: https://doi.org/10.5281/zenodo.3730897 ^19^.

License: MIT license
