# Supplementary material for: Making health economic models Shiny: A tutorial
Source: Wellcome Open Res. 2020 Jul 31;5:69. Originally published 2020 Apr 14. [Version 2] doi: 10.12688/wellcomeopenres.15807.2 (PMC7459889; doi:10.12688/wellcomeopenres.15807.2)
Supplement: Peer review history

# Peer review: Reviewer response for version 2

**DOI:** 10.21956/wellcomeopenres.17775.r39795

**Published:** 2020-08-28

**Authors:** Talitha L. Feenstra

**Affiliations:** 1 Groningen Research Institute of Pharmacy, University of Groningen, Groningen, The Netherlands

I agree with the changes. Thanks to the authors for a clear reply to comments made.

Is the rationale for developing the new method (or application) clearly explained?

Yes

Is the description of the method technically sound?

Partly

Are the conclusions about the method and its performance adequately supported by the findings presented in the article?

Yes

If any results are presented, are all the source data underlying the results available to ensure full reproducibility?

Yes

Are sufficient details provided to allow replication of the method development and its use by others?

Yes

Reviewer Expertise:

NA

I confirm that I have read this submission and believe that I have an appropriate level of expertise to confirm that it is of an acceptable scientific standard.

# Peer review: Reviewer response for version 2

**DOI:** 10.21956/wellcomeopenres.17775.r39794

**Published:** 2020-08-03

**Authors:** Yiqiao Xin

**Affiliations:** 1 Health Economics and Health Technology Assessment, Institute of Health and Wellbeing, University of Glasgow, Glasgow, UK

The authors have sufficiently addressed my comments. Thank you for producing this tutorial and I believe it will be very useful to its audience. I have no further comments to make.

Is the rationale for developing the new method (or application) clearly explained?

Yes

Is the description of the method technically sound?

Yes

Are the conclusions about the method and its performance adequately supported by the findings presented in the article?

Yes

If any results are presented, are all the source data underlying the results available to ensure full reproducibility?

Yes

Are sufficient details provided to allow replication of the method development and its use by others?

Yes

Reviewer Expertise:

Trial and model based economic evaluations, network meta-analysis, outcome research, development of Shiny apps.

I confirm that I have read this submission and believe that I have an appropriate level of expertise to confirm that it is of an acceptable scientific standard.

# Peer review: Reviewer response for version 1

**DOI:** 10.21956/wellcomeopenres.17335.r39194

**Published:** 2020-07-10

**Authors:** Yiqiao Xin

**Affiliations:** 1 Health Economics and Health Technology Assessment, Institute of Health and Wellbeing, University of Glasgow, Glasgow, UK

This paper provides a tutorial for those that have developed state transition models in R to be able to develop a Shiny interface allowing stakeholders to scrutinize the assumptions. Overall this is a well written paper for its specific audience, especially in the context of an increasing trend of developing models in R. it also pointed to some useful references for people who are interested in developing state transition models in R at first place, as well as those who have some experience in R but want to build an interface. I particularly like figure 1 which is a very nice diagram depicting the relationship between the ui.R and server.R.

 Please find my comments as below.

1. In the Introduction, it will be useful to clarify what audience this paper is intended to. Clearly this is not a tutorial for those people who never used R to develop models.
2. In the Methods, the PSA section complicates the purpose of this tutorial which is teaching people how to build a Shiny app for their models. Putting all the functions here in the main text makes the tutorial look daunting than it should be. If the authors have to keep it, probably consider moving it to appendix, or later on in the text, if the interface has an input into the PSA. Afterall, my understanding is that this paper is not teaching people how to build a state transition model and PSA in R.
3. The authors mentioned a few times the use of ggplots/plotly or other programming languages to make the interface aesthetically pleasing. For the benefit of users, can the authors provide an example of an economic model app that embedded the codes that provide aesthetically pleasing interface? if they don’t have one, it will be helpful if they could reference a few existing Shiny apps for EE models that incorporated these features and have codes openly available.
4. The discussion section could be improved by further discussing the following points:

      a. Can this tutorial be scaled well to other types of economic models, e.g. Discrete event simulation? It will be helpful to point the differences in building the ui and server when applied to other type of models, and discuss the issues and caveats.

        

        b. The authors could further discuss the usefulness of a Shiny interface for decision makers. Although Shiny can achieve some level of visualisation of the results making it easier for decision makers to examine the impact on the results when conducting sensitivity analysis, decision makers can’t use it to further check the essential calculations and relationships between the parameters. In other words, If decision makers have the knowledge of R coding, they may not need to the Shiny interface to help visualise, whereas if they lack the knowledge of R coding, it is still a barrier for them to check the fundamental structure of model which are coded in the server.R file.

         c. Does the Shiny app encounter capacity issues when running a large complex economic model, especially when running PSA, or value of sample information? In our experience, a Shiny app running complex simulations is memory demanding and developers will need to purchase the plans provided by R Studio to be able to run on the server after publishing. It will be useful for the authors to provide some suggestions or solutions if they have similar experiences. 

   

 Minor points:

1. There are some page numbers in bracket in the manuscript – is this required by the journal? some of them are not books but journal articles, and in some places where a journal article was cited, these page numbers are not shown.
2. Page 7, after figure 2, first paragraph, “The title panel contains the title “Sick Sicker Model….. and an Action Button (“Run/update model”).” Please be consistent in terms of upper/lower case when referring to the input widget, i.e. “action button”.
3. Same paragraph as above, line 4, it said ‘Note that this is an addition of the coding framework provided by Alarid-Escudro et al. , (2019)’. Not sure why this sentence is put here, perhaps consider moving it to somewhere else as it seems can be applied to the whole Shiny app development. Also, there is an extra ‘,’ at the end of the sentence.
4. Page 8, ‘ Creating the server function’ section, third paragraph, line 2/3: “The function *renderTable* rerenders the table continuously so that the table always reflects the values …” Calling it ‘rerender’ is a little confusing when describing the function 'renderTable'. It just means it updates the output table each time after the input is updated by the end user.
5. Page 9, in the Server.R codes, ‘# when action button pressed ... observeEvent(input$run_model,  ignoreNULL = F, {…”, what does the part ‘ignoreNULL = F’ do?
6. In the discussion, it will be useful to provide a reference for the WHO Shiny app.

Is the rationale for developing the new method (or application) clearly explained?

Yes

Is the description of the method technically sound?

Yes

Are the conclusions about the method and its performance adequately supported by the findings presented in the article?

Yes

If any results are presented, are all the source data underlying the results available to ensure full reproducibility?

Yes

Are sufficient details provided to allow replication of the method development and its use by others?

Yes

Reviewer Expertise:

Trial and model based economic evaluations, network meta-analysis, outcome research, development of Shiny apps.

I confirm that I have read this submission and believe that I have an appropriate level of expertise to confirm that it is of an acceptable scientific standard.

# Response

**Published:** 2020-07-22

**Authors:** Robert Smith

**Affiliations:** University of Sheffield, UK

*Thank-you for reviewing this paper and for your constructive comments, we have taken both reviewers’ comments into account and tried to make changes where possible to improve the paper. I provide responses to the comments one-by-one below:*

**R1: This paper provides a tutorial for those that have developed state transition models in R to be able to develop a Shiny interface allowing stakeholders to scrutinize the assumptions. Overall this is a well written paper for its specific audience, especially in the context of an increasing trend of developing models in R. it also pointed to some useful references for people who are interested in developing state transition models in R at first place, as well as those who have some experience in R but want to build an interface. I particularly like figure 1 which is a very nice diagram depicting the relationship between the ui.R and server.R.**

****

*Thank-you, we hope it is useful for others trying to improve model transparency and aim to build further teaching resources around it.*

****

****

**R1: In the Introduction, it will be useful to clarify what audience this paper is intended to. Clearly this is not a tutorial for those people who never used R to develop models.**

*We have changed the text to specifically mention this, we have added the following sentence to the last paragraph of the introduction section:  “This paper aims to provide a tutorial for those familiar with decision modelling in R to create web based user interfaces for R models using R Shiny.”*

**R1: In the Methods, the PSA section complicates the purpose of this tutorial which is teaching people how to build a Shiny app for their models. Putting all the functions here in the main text makes the tutorial look daunting than it should be. If the authors have to keep it, probably consider moving it to appendix, or later on in the text, if the interface has an input into the PSA. Afterall, my understanding is that this paper is not teaching people how to build a state transition model and PSA in R.**

*There is a conflict here between what different people may want from the paper (the other reviewer wanted more detail on this function). The aim was to step the reader through this example in detail, and since this deviates from the Sick-Sicker resources provided by DARTH we think it should remain in the paper. We would have ideally put this in the appendix but Wellcome Open Research does not allow appendices.*

**The authors mentioned a few times the use of ggplots/plotly or other programming languages to make the interface aesthetically pleasing. For the benefit of users, can the authors provide an example of an economic model app that embedded the codes that provide aesthetically pleasing interface? if they don’t have one, it will be helpful if they could reference a few existing Shiny apps for EE models that incorporated these features and have codes openly available.**

*To address this ee have added examples in the text of some existing shiny applications that we feel are well designed. In particular a lot of thought has been put into BresMed’s InterRface (described in Rose Hart’s recent paper referenced in this amendment). However the code for the InteRface tool has not been made open-source.*

**Can this tutorial be scaled well to other types of economic models, e.g. Discrete event simulation? It will be helpful to point the differences in building the ui and server when applied to other type of models, and discuss the issues and caveats.**

****

*We have added the following text to the discussion section, but in short, yes… and we hope to do so in the future but for this paper we aimed for simplicity.*

*“It is worth noting that the simple model chosen as an exemplar is a markov model, however the method described can be applied to any model built using R, regardless of model type. For example in this case, the f_MM_sicksicker function could be replaced by a function containing any type of model (e.g. a DES model).”*

**The authors could further discuss the usefulness of a Shiny interface for decision makers. Although Shiny can achieve some level of visualisation of the results making it easier for decision makers to examine the impact on the results when conducting sensitivity analysis, decision makers can’t use it to further check the essential calculations and relationships between the parameters. In other words, If decision makers have the knowledge of R coding, they may not need to the Shiny interface to help visualise, whereas if they lack the knowledge of R coding, it is still a barrier for them to check the fundamental structure of model which are coded in the server.R file.**

****

*We added the following paragraphs in the discussion section to clarify this important issue and better explain the benefits (and limitations) of using R shiny applications to communicate health economic decision models:*

*A well designed user interface can allow users to explore and better understand the relationship between model input and results. This allows users to tailor the health economic model to their specific situation and assumptions, without the expense of creating a new model. This may be  particularly useful in the following scenarios: Firstly, in areas where one health economic decision model is applied in range of circumstances (e.g. in public health, models are often built to be used in a number of different countries). Secondly, when the full model source code can or may not be shared (e.g. for proprietary or privacy reasons). R shiny apps can be made available in a way that allows users to interact with the web interface, without revealing the model behind it. Finally, R shiny apps may enable stakeholders and decision makers, who would otherwise not be able to interact directly with statistical computer models, to experiment with and to reflect on various scenarios and the validity of model inputs and outputs.*

*However, in all of these scenarios, the ability for users to test different assumptions is not without limits: the available options to vary point estimates or the uncertainty around input parameters are defined by the model developer, and it is also not possible to specify alternative model structures or test any other aspect that the developer did not implement. Therefore the model source code and data will still need to be made available to reviewers to allow for a thorough assessments of health economic models. Further investigation into how to communicate economic decisions models in a transparent and inclusive way is an important avenue of future research.*

**Does the Shiny app encounter capacity issues when running a large complex economic model, especially when running PSA, or value of sample information? In our experience, a Shiny app running complex simulations is memory demanding and developers will need to purchase the plans provided by R Studio to be able to run on the server after publishing. It will be useful for the authors to provide some suggestions or solutions if they have similar experiences.**

****

*Again, a really useful point. We have never had an issue running a model remotely with ShinyIO… It generally seems as fast or faster than a moderately expensive laptop. We know others use it for PSA/VOI so suspect that the servers would be able to handle most models. That being said we have never tried to do anything super-computationally burdensome - for this other providers used to dealing with big data/big computation (e.g. Amazon Web Services) may be more suitable. We have added the below text:*

**

*“Server specifications should be chosen to match model requirements: while simple Markov chain state transition models may run on almost any server, more computationally burdensome models e.g. agent-based models may require considerable computing power”*

****

**There are some page numbers in bracket in the manuscript – is this required by the journal? some of them are not books but journal articles, and in some places where a journal article was cited, these page numbers are not shown.**

*These are correct – they only apply to the print version of the documents, they are on the published paper PDF versions.*

****

**Page 7, after figure 2, first paragraph, “The title panel contains the title “Sick Sicker Model….. and an Action Button (“Run/update model”).” Please be consistent in terms of upper/lower case when referring to the input widget, i.e. “action button”.**

*Thank-you for this, our mistake, now corrected in the text to all be lower case.*

****

**Same paragraph as above, line 4, it said ‘Note that this is an addition of the coding framework provided by Alarid-Escudro et al., (2019)’. Not sure why this sentence is put here, perhaps consider moving it to somewhere else as it seems can be applied to the whole Shiny app development. Also, there is an extra ‘,’ at the end of the sentence.**

*We have moved this to the top of the methods section. Thank-you this is much clearer.*

**Page 8, ‘ Creating the server function’ section, third paragraph, line 2/3: “The function renderTable rerenders the table continuously so that the table always reflects the values …” Calling it ‘rerender’ is a little confusing when describing the function 'renderTable'. It just means it updates the output table each time after the input is updated by the end user.**

*We have simplified this paragraph to read:  “The function renderTable generates a table from the model results to display it on the web interface.”*

****

**Page 9, in the Server.R codes, ‘# when action button pressed ... observeEvent(input$run_model,  ignoreNULL = F, {…”, what does the part ‘ignoreNULL = F’ do?**

*Apologies we should have explained, we have added “Setting the parameter ignoreNULL to False lets the model run when it is initialised, i.e. when the app is started.”  to that paragraph.*

****

**In the discussion, it will be useful to provide a reference for the WHO Shiny app.**

*Thanks we have added a reference to this.*

# Peer review: Reviewer response for version 1

**DOI:** 10.21956/wellcomeopenres.17335.r38662

**Published:** 2020-07-01

**Authors:** Talitha L. Feenstra

**Affiliations:** 1 Groningen Research Institute of Pharmacy, University of Groningen, Groningen, The Netherlands

This tutorial clearly explains the use of R shiny to present results from HE decision models to a general audience, allowing them to change several input parameters and see the effects on the model outcomes. 

 The text and R code that comes along with it on GitHub is mostly clear. I tried the code and it worked without any issues after downloading. With just the paper in hand that would have been somewhat more difficult, since parts of code are presented and it is not fully clear how they link together.

 The sourcing part is not complete e.g.

 Overall this tutorial is a nice illustration and helpful if you want to start working with HE models using Shiny. However the example is strongly simplified, up to the unrealistic (an intervention which does not affect transition rates).

 Since it is a tutorial, this paper has no results section.

 In the introduction maybe it is relevant to refer to actual existing applied HE decision models in R with Shiny interface (e.g.http://dismod.ndph.ox.ac.uk/kidneymodel/app/, https://github.com/InnovationValueInitiative, both with publications available:

 Pharmacoeconomics 2019 ;37(6):829-843. doi: 10.1007/s40273-018-00765-2. A Flexible Open-Source Decision Model for Value Assessment of Biologic Treatment for Rheumatoid Arthritis, D Incerti, JR Curtis et al ^1^.

 Heart. 2017;103(23):1880-1890.

 doi: 10.1136/heartjnl-2016-310970. A Policy Model of Cardiovascular Disease in Moderate-To-Advanced Chronic Kidney Disease. I Schlackow, S Kent et al ^2^. , I guess more will exist)

 The argumentation/opinion that the future of health economics is in automated click and run models is not at its place in the methods section. This should go to the discussion and be more thoroughly supported by arguments. I wonder how conceptual model selection and input data analysis to fill a HE decision model could be really performed using a pre-specified framework. Surely this framework should  be very flexible. It also seems to prohibit involvement of stakeholders in decisions concerning model structure and input data analysis/interpretation. 

- How will the problem of structural uncertainty be addressed by models made available to HE decision makers and other users to perform their own uncertainty analysis? The text in the paper can improve from further clarification at some points.
- The PSA function is not explained in the paper and this is the new part compared to the SickSicker publication. So I would rather have expected some explanation on this (and maybe less on the SickSicker, which is already published/explained)
- What distributions were chosen for what model parameters? How could modelers apply this code as a basis for adding a PSA into their own application and have it serve as input for a Shiny? Ideally: the user would have the option to choose some of the PSA settings other than just the number of runs, e.g. the type of distribution and/or the parameters in this distribution.
- The initial set-up part is unclear, and the code is not the same as the R code available online.
- The discussion refers to "inform teaching content", what teaching is meant here? And whom are taught?

Minor unclarities:

 Using cost to refer to intervention cost is confusing. It also presumes the control has zero costs. It is not clear whether the intervention cost is one-off or annually. But that might be explained in the SickSicker paper.

 Using numerous for the number of available packages and then referring to three seems a bit overdone.

 Health economics is more than HE decision modeling, I would refer to HE decision modeling, or cost-effectiveness analysis rather than health economics which is a very broad field.

- For some jurisdictions it would be functional to allow for two different discount rates (on costs and on health outcomes).
- The app uses limits for the axes in the graph that move with numbers provided. This is of course needed when users are unrestricted in the values they provide regarding intervention costs, however as a result, seeing the results of having different intervention costs, requires proper attention for the values at the y-axis, which change with every new value provided by the user. It would be more informative to see the c/e cloud move rather than the axis scale. Maybe that could be solved by having a range for the intervention costs pre-specified (or alternatively let the axis limits depend on the treatment costs more like a step function).

Is the rationale for developing the new method (or application) clearly explained?

Yes

Is the description of the method technically sound?

Partly

Are the conclusions about the method and its performance adequately supported by the findings presented in the article?

Yes

If any results are presented, are all the source data underlying the results available to ensure full reproducibility?

Yes

Are sufficient details provided to allow replication of the method development and its use by others?

Yes

Reviewer Expertise:

Health economics, specifically HE decision modeling

I confirm that I have read this submission and believe that I have an appropriate level of expertise to confirm that it is of an acceptable scientific standard, however I have significant reservations, as outlined above.

## References

[1] : A Flexible Open-Source Decision Model for Value Assessment of Biologic Treatment for Rheumatoid Arthritis. *Pharmacoeconomics*.37(6) : 10.1007/s40273-018-00765-2 829-843 10.1007/s40273-018-00765-2 30737711

[2] : A policy model of cardiovascular disease in moderate-to-advanced chronic kidney disease. *Heart*.103(23) : 10.1136/heartjnl-2016-310970 1880-1890 10.1136/heartjnl-2016-310970 28780579PMC5749372

# Response

**Published:** 2020-07-22

**Authors:** Robert Smith

**Affiliations:** University of Sheffield, UK

Thank-you for taking the time to review this paper, the feedback is much appreciated and we hope the new version addresses all of your comments.

**R2: This tutorial clearly explains the use of R shiny to present results from HE decision models to a general audience, allowing them to change several input parameters and see the effects on the model outcomes.**

*Thank you, we are glad it was useful and clear.*

**R2: The text and R code that comes along with it on GitHub is mostly clear. I tried the code and it worked without any issues after downloading. With just the paper in hand that would have been somewhat more difficult, since parts of code are presented and it is not fully clear how they link together.**

*This is a difficult issue since preferences for code in paper vary from person to person (e.g. between yourself and the other reviewer). Unfortunately Wellcome Open Research does not allow appendices so we therefore did not provide all the R model code in the paper. The full, commented version of the code is available on the Zenodo data repository.*

**R2: The sourcing part is not complete e.g.**

*I’m not sure what you mean, when we checked the two were the same, we are happy to make any changes where inconsistencies exist.*

**R2: Overall this tutorial is a nice illustration and helpful if you want to start working with HE models using Shiny. However the example is strongly simplified, up to the unrealistic (an intervention which does not affect transition rates).**

**Since it is a tutorial, this paper has no results section.**

*This example takes an existing model used as a teaching tool by the DARTH group. We did this because many people have learnt R for HTA from their courses so it made sense to extend upon this.*

**R2: The argumentation/opinion that the future of health economics is in automated click and run models is not at its place in the methods section. This should go to the discussion and be more thoroughly supported by arguments. I wonder how conceptual model selection and input data analysis to fill a HE decision model could be really performed using a pre-specified framework. Surely this framework should  be very flexible. It also seems to prohibit involvement of stakeholders in decisions concerning model structure and input data analysis/interpretation.**

*Agreed – we removed the sentence, and don’t feel an elaboration in the discussion section to be of additional value.*

**R2: How will the problem of structural uncertainty be addressed by models made available to HE decision makers and other users to perform their own uncertainty analysis? The text in the paper can improve from further clarification at some points.**

*We completely agree, a similar point was raised by the other reviewer. We have added the below section to the discussion. We hope that this clarifies things:*

*A well designed user interface can allow users to explore and better understand the relationship between model input and results. This allows users to tailor the health economic model to their specific situation and assumptions, without the expense of creating a new model. This may be  particularly useful in the following scenarios: Firstly, in areas where one health economic decision model is applied in range of circumstances (e.g. in public health, models are often built to be used in a number of different countries). Secondly, when the full model source code can or may not be shared (e.g. for proprietary or privacy reasons). R shiny apps can be made available in a way that allows users to interact with the web interface, without revealing the model behind it. Finally, R shiny apps may enable stakeholders and decision makers, who would otherwise not be able to interact directly with statistical computer models, to experiment with and to reflect on various scenarios and the validity of model inputs and outputs.*

*However, in all of these scenarios, the ability for users to test different assumptions is not without limits: the available options to vary point estimates or the uncertainty around input parameters are defined by the model developer, and it is also not possible to specify alternative model structures or test any other aspect that the developer did not implement. Therefore the model source code and data will still need to be made available to reviewers to allow for a thorough assessments of health economic models. Further investigation into how to communicate economic decisions models in a transparent and inclusive way is an important avenue of future research.*

**R2: The PSA function is not explained in the paper and this is the new part compared to the SickSicker publication. So I would rather have expected some explanation on this (and maybe less on the SickSicker, which is already published/explained).**

*This point contradicted the preference of the other reviewer, but in fact we agree with you on this one. We have added much more detail on the PSA function (but kept the existing detail for the SickSicker).*

**R2: What distributions were chosen for what model parameters? How could modelers apply this code as a basis for adding a PSA into their own application and have it serve as input for a Shiny? Ideally: the user would have the option to choose some of the PSA settings other than just the number of runs, e.g. the type of distribution and/or the parameters in this distribution.**

*We state that the function “returns a data-frame of probabilistic sensitivity analysis inputs: transition probabilities between health states using a beta distribution, hazard rates using a log-normal distribution, costs using a gamma distribution and utilities using a truncnormal distribution”.*

**R2: The initial set-up part is unclear, and the code is not the same as the R code available online.**

*Thank-you, we have updated the paper to match the Zenodo repository.*

**R2: The discussion refers to "inform teaching content", what teaching is meant here? And whom are taught?**

*Thanks- changed to read: “Finally, a challenge (and opportunity) exists to create user-interfaces that are most user-friendly for decision makers in this field; this is an area of important research that requires closer collaboration between decision makers, stakeholders and health economic decision model developers.”*

**R2: Using cost to refer to intervention cost is confusing. It also presumes the control has zero costs. It is not clear whether the intervention cost is one-off or annually. But that might be explained in the SickSicker paper.**

*“To clarify the issue we have revised the comment # cost of treatment to #cost of intervention treatment in states sick & sicker.”*

**R2: Using numerous for the number of available packages and then referring to three seems a bit overdone.**

*I was slightly unsure what you mean, but we have changed the word numerous for “different”.*

**R2: Health economics is more than HE decision modeling, I would refer to HE decision modeling, or cost-effectiveness analysis rather than health economics which is a very broad field.**

*The sentence you may be referring to (‘we believe this is the future of health economics’) was removed from the paper in response to a comment from the other reviewer In other instances, we changed ‘health economic models’ to ‘health economic decision models’, if appropriate.*

*We also changed ‘health economists’ to ‘health economic modellers’ e.g. (“The first is the challenge of up-skilling health economic modellers used to working in Microsoft Excel.”)*

**R2: For some jurisdictions it would be functional to allow for two different discount rates (on costs and on health outcomes).**

*This is an interesting conceptual issue for cost-effectiveness modelling in general, but it doesn't seem within the scope of this paper on the use of R/R shiny for HE modelling.*

**R2: The app uses limits for the axes in the graph that move with numbers provided. This is of course needed when users are unrestricted in the values they provide regarding intervention costs, however as a result, seeing the results of having different intervention costs, requires proper attention for the values at the y-axis, which change with every new value provided by the user. It would be more informative to see the c/e cloud move rather than the axis scale. Maybe that could be solved by having a range for the intervention costs pre-specified (or alternatively let the axis limits depend on the treatment costs more like a step function).**

*We decided not to have a limit for the axes, or use any other additional style element, to provide a ‘minimal working code example’. This is common practice in coding tutorials. A more sophisticated application could/should use a Plotly function to create an interactive plot, but this would still change depending on the range of values generated by the PSA.*
